# Sparse feature selection for classification and prediction of metastasis in endometrial cancer

**DOI:** 10.1186/s12864-017-3604-y

**Published:** 2017-03-27

**Authors:** Mehmet Eren Ahsen, Todd P. Boren, Nitin K. Singh, Burook Misganaw, David G. Mutch, Kathleen N. Moore, Floor J. Backes, Carolyn K. McCourt, Jayanthi S. Lea, David S. Miller, Michael A. White, Mathukumalli Vidyasagar

**Affiliations:** 1 IBM Research, Yorktown Heights, NY USA; 2 The University of Tennessee, College of Medicine, KnoxvilleTN, USA; 3 Apple R&D, Austin, TX USA; 4 Harvard University, Cambridge, MA USA; 5 The Washington University School of Medicine, St. Louis, MO USA; 6 The University of Oklohoma, Norman, OK USA; 7 The Ohio State University, Columbus, OH USA; 8 Women and Infants Hospital, Brown University, Providence, RI, USA; 9 University of Texas Southwestern Medical Center, TX, Dallas, USA; 10 The University of Texas at Dallas, Richardson, TX USA

**Keywords:** Endometrial cancer, Lymph node metastasis, Sparse classification, Machine learning

## Abstract

**Background:**

Metastasis via pelvic and/or para-aortic lymph nodes is a major risk factor for endometrial cancer. Lymph-node resection ameliorates risk but is associated with significant co-morbidities. Incidence in patients with stage I disease is 4–22% but no mechanism exists to accurately predict it. Therefore, national guidelines for primary staging surgery include pelvic and para-aortic lymph node dissection for all patients whose tumor exceeds 2cm in diameter. We sought to identify a robust molecular signature that can accurately classify risk of lymph node metastasis in endometrial cancer patients. 86 tumors matched for age and race, and evenly distributed between lymph node-positive and lymph node-negative cases, were selected as a training cohort. Genomic micro-RNA expression was profiled for each sample to serve as the predictive feature matrix. An independent set of 28 tumor samples was collected and similarly characterized to serve as a test cohort.

**Results:**

A feature selection algorithm was designed for applications where the number of samples is far smaller than the number of measured features per sample. A predictive miRNA expression signature was developed using this algorithm, which was then used to predict the metastatic status of the independent test cohort. A weighted classifier, using 18 micro-RNAs, achieved 100% accuracy on the training cohort. When applied to the testing cohort, the classifier correctly predicted 90% of node-positive cases, and 80% of node-negative cases (FDR = 6.25%).

**Conclusion:**

Results indicate that the evaluation of the quantitative sparse-feature classifier proposed here in clinical trials may lead to significant improvement in the prediction of lymphatic metastases in endometrial cancer patients.

**Electronic supplementary material:**

The online version of this article (doi:10.1186/s12864-017-3604-y) contains supplementary material, which is available to authorized users.

## Background

Endometrial cancer (adenocarcinoma of the uterine corpus) is the most common malignancy unique to women. It is estimated that in 2016, 60,500 women will develop endometrial cancer and 10,470 will die of it [1]. A major risk factor is metastasis via pelvic and/or para-aortic lymph nodes. For patients with cancer confined to the uterus, the five-year recurrence-free survival is 93%. However, metastasis to pelvic lymph nodes and/or to aortic lymph nodes decreases this to 57.8% and 41.2% respectively [2]. In consequence, primary staging surgery for endometrial cancer often consists of removal of the uterus, ovaries, fallopian tubes, and pelvic and para-aortic lymph node dissection. Morbidities associated with lymph node dissection include increased operative times, increased blood loss, ileus, increased number of thromboembolic events, lymphocyst formation, and major wound dehiscence, all of which adversely affect the patients’ health and quality of life [3].

Incidence of pelvic and para-aortic node metastasis in patients with stage I endometrial cancer varies from 4–22% depending on grade, depth of invasion, lymphovascular space invasion, and histologic subtype [4]. Patients harboring tumors less than 2 centimeters in diameter and with less than 50% myometrial invasion are considered to be at low risk for lymphatic metastasis [5]. In a key clinical study, patients whose tumors violate these criteria were recommended for lymphadenectomy. Yet, within this high risk group, only 22% had lymph node metastasis, suggesting that 78% of the lymphadenectomies were unnecessary [5]. A more recent study [6] that separately considered pelvic versus paraaortic lymph node invasion showed little improvement in this statistic. It is therefore clear that current best practice clinical-pathologic parameters are grossly insufficient for reasonable prediction of metastatic disease [5].

To address this clinical need, efforts have been made to develop molecular signatures for predicting lymph node metastasis. An ideal classifier should consist of two parts: a set of features that are highly predictive, and a numerical procedure for combining the measured values of these features so as to make a binary prediction (yes or no) about the metastatic risk of a patient. Most of the current molecular signatures under consideration perform the first step but not the second; that is, they contain a set of key biomarkers, but do not apply a systematic method for predicting the outcome on an independent testing cohort. For example, absence of expression of the estrogen receptor (ER) and progesterone receptor (PR) genes, the so-called double-negative situation, correlates with increased risk of lymph node metastasis [7]. However, this correlation does not appear to translate into a prognostic test that can predict lymph node metastasis on a patient-by-patient basis. Of note, levels of CA125 together with three parameters obtained from radiological images are sufficient to correctly identify low-risk patients. However, about half of patients are incorrectly classified as at risk for metastasis [8]. Finally, CA125 together with HE4 are positively correlated with tumor grade as well as risk of lymph node metastasis, but again, this correlation has not been translated into a prognostic classifier [9].

Machine learning is a discipline that combines engineering, statistics, and computer science that can potentially be used to generate highly informative biomarkers automatically from biological data sets. Most effective and widely used machine learning methods, such as the support vector machine (SVM) [10], are specialized for applications where the number of samples is far larger than the number of features per sample. However, a common conundrum for medical research applications is that the number of characterized patient samples is far smaller than the number of molecular measurements (features) per sample. Consequently, the application of machine learning methods to translational medical research must address two distinct but interwoven challenges: the selection of a handful of the most predictive features from a very large initial set of features, and a method for combining the measured values of these predictive features into a numerical recipe for making predictions. Motivated by this consideration, we have developed an algorithm that is specifically tailored for such biological applications. The *ℓ*_1_-norm SVM formulated in [11] guarantees sparse solutions, but in biological applications where the data is highly correlated its prediction performance is poor and the set of nonzero features is not stable when the data is noisy. To overcome this limitation, the Elastic Net (EN) algorithm was introduced in [12], which minimizes a convex combination of the *ℓ*_1_-norm and the *square* of the *ℓ*_2_-norm. It is shown in [12, Theorem 1] that the EN formulation achieves the so-called “grouping effect,” whereby highly correlated features are achieved near equal weight. However, in a theoretical paper written by a subset of the present authors [13], it is established that the EN formulation is not suitable for compressed sensing; see [13, Theorem 2.1]. To overcome these limitations, we take a novel approach and use a convex combination of *ℓ*_1_- and *ℓ*_2_-norms in our sparse classification algorithm.

In [13], it is established that our algorithm out-performs both the *ℓ*_1_-norm SVM and the Elastic Net. Robustness to variations in experimental protocols is achieved by incorporating recursive feature elimination [14], and stability selection [15]. When applied to quantitative genome-scale microRNA expression data from 86 clinically annotated primary endometrial tumors, 18 micro-RNAs were recovered that are sufficient to predict the risk of lymph node metastasis within the training cohort. This biomarker panel was tested on an independent cohort of 28 tumors, and returned predictions with high sensitivity, low false discovery rate, and *P*<0.0004. The panel therefore provides a path towards the development of a practical molecular diagnostic to avoid unnecessary surgeries (and their associated morbidities) in patients who are not at risk currently about 78% of all lymph node resections for endometrial cancer patients in the USA. This study is thus a transdisciplinary combination of two distinct advances: (i) a new algorithm for sparse feature selection in binary classification problems, and (ii) its application to predict the risk of metastasis in endometrial cancer.

## Results

### Selection of training cohort and generation of the predictive feature matrix

We established strict inclusion and exclusion criteria for this study in an effort to control for the known clinical factors associated with lymph node metastasis. Specifically, we excluded all non-endometrioid histologies as these tumors are clinically and biologically distinct from the more common endometrioid histologic subtypes and are much more likely to show evidence of lymphatic spread. We also excluded those tumors with gross evidence of extra-uterine disease at the time of surgery, thus limiting inclusion to clinical stage I tumor, as the presence of gross pelvic or intra-abdominal tumor increases the likelihood of positive lymph node metastasis [3]. Fifty stage I (1988 FIGO staging) and 50 stage IIIC frozen endometrial cancer samples were obtained from the Gynecologic Oncology Group tumor bank according to the above criteria. The samples were collected from patients enrolled in GOG tissue acquisition protocol 210 which established a repository of clinical specimens with detailed clinical and epidemiologic data from patients with surgically staged endometrial carcinoma. Samples were matched for age, grade, presence of lymphvascular space invasion, and where possible for race. All patients enrolled in GOG 210 have undergone comprehensive surgical staging consisting of total abdominal hysterectomy, bilateral salpingo-oophorectomy, pelvic and para-aortic lymphadenectomy. All patients included in this study had no gross or pathologic evidence of extra-uterine disease aside from lymph node metastasis and could therefore be considered clinical stage I tumors. While more patients with stage IIIC tumors had LVSI and deep myometrial invasion relative to the stage I tumors, the majority of patients in both groups had poor prognostic factors and would have been included in the high-intermediate risk (HIR) subgroup set forth in GOG protocol 99 [16]. Specifically, 65% the stage I tumors and 81% of the stage IIIC tumors would be considered HIR (*P*=0.5), highlighting the homogeneity of the entire tumor set (Table 1). All tumors were subjected to central pathologic review by the GOG.

**Table 1:** Clinical Parameters of the training cohort

		Lymph node	Lymph node
		negative (*n*=46)	positive (*n*=47)
Age	≤ 60	23 (50%)	19 (40%)
	>60	23 (50%)	28 (60%)
Race	AA	2 (4%)	3 (6%)
	non-AA	44 (96%)	44 (94%)
Tumor Grade (*n*=93)	1	8 (17%)	13 (28%)
	2	14 (30%)	17 (36%)
	3	24 (53%)	17 (36%)
LVSI (*n*=92)	Present	17 (38%)	37 (80%)
	Absent	28 (62%)	9 (20%)
Myometrial Invasion	Inner 1/2	23 (50%)	11 (25%)
(*n*=90)	Outer 1/2	23 (50%)	33 (75%)

Quantitative measurement of miRNA expression was chosen for detection of putative predictive features. As a family, miRNAs represent a relatively compact feature set which is, never-the-less, profoundly integrated with cell and tissue behavior [17–19]. Moreover, miRNA expression patterns have been identified that can predict benign vs. malignant disease, histologic subtypes, survival, and response to chemotherapy [20–22]. Two recent surveys highlight the role of miRNAs in cancer in general [23] and endometrial cancer in particular [24].

**Fig. 1:**
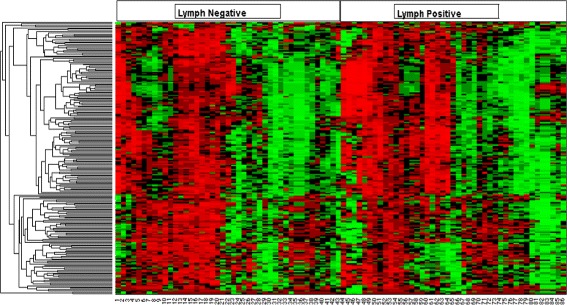
Hierarchical clustering of training data. Unsupervised two-way hierarchical clustering of the 213 miRNA expression levels across the 86 tumor samples. The 43 samples at left are lymph node-negative while the 43 samples at right are lymph node-positive. It is evident that there is no discernible pattern in the clustering

Total cellular miRNA was extracted from all tissues and measured using LNA-based detection arrays (Additional file 1: Table S1). 86 samples passed quality controls based on RNA integrity and expression array performance. Among the 1,428 available probe sets, 213 miRNAs were detectable in all 86 samples (Additional file 1: Table S2). An unsupervised two-way hierarchical clustering of the resulting miRNA expression values within each subclass revealed substantial expression variation between tumors, with no qualitatively evident distinctions between subclasses (Fig. 1).

### Generation of molecular signature for predicting lymph node metastasis

In order to detect candidate quantitative microRNA feature sets within the primary tumors that may discriminate between node positive and node negative disease, as well as a numerical procedure for combining the measured values of the features, we turned to machine learning protocols. When the number of features is larger than the number of samples, which is typical for biological problems such as the one here, machine learning approaches commonly encounter a phenomenon known as “over-fitting,” wherein a classifier does an excellent job on the training data, but has poor generalization abilities. To overcome this problem, we developed a sparse classification algorithm that uses a convex combination of *ℓ*_1_- and *ℓ*_2_ norms as a regularization term in its objective function.

The traditional support vector machine (SVM), as broadly applied to medical research, generates a classifier via a so-called discriminant function, which is a weighted linear combination of the measured values of the features, minus a threshold. If the discriminant value associated with a particular sample is positive, the sample is assigned to the positive class (in our case, at risk of metastasis), and is assigned to the negative class otherwise (in our case, not at risk). The main drawback of the traditional SVM is that in general the discriminant assigns a nonzero weight to all the features, which is unacceptable when the number of features is large. Therefore we replaced the Euclidean or *ℓ*_2_-norm distance measure used in the traditional SVM algorithm by a combination of the *ℓ*_2_-norm and the so-called *ℓ*_1_-norm, which is the sum of the absolute values of a vector. The use of *ℓ*_1_-norm in the penalty function causes the classifier to be sparse, while the *ℓ*_2_-norm causes correlated features to be selected together providing robustness to the method; see [13, Theorem 2.2]. To reduce the size of the feature set still further, we applied recursive feature elimination (RFE) [14]. When RFE is applied with the traditional SVM, the performance is often erratic, and the algorithm must be iterated many times before a satisfactory result can be obtained, if at all [14]. However, because the combined *ℓ*_1_-and *ℓ*_2_-norm SVM assigns “exactly" zero weights to several features at once, RFE together with the combined *ℓ*_1_- and *ℓ*_2_-norm SVM led to a steady improvement in the fitting at each iteration. Finally, to ensure that the chosen feature set is relatively insensitive to noise, at each iteration of lone star we divided the available samples into random training and cross-validation sets, repeated this exercise many times till the number of the selected features stabilizes. This approach is known as “stability selection” [15]. The number of such divisions is the only user defined parameter in lone star and in practice we have observed that 80 iterations is optimal, in the sense that increasing this number does not lead to better performance. Furthermore, to avoid over-fitting lone star compensates for it automatically by increasing the number of iterations. The overall algorithm is referred to in its full form as “ *ℓ*_1_-, *ℓ*_2_-norm SVM *t*-test and RFE,” or “lone star” for short. To facilitate its use by the general community, a Matlab implementation of the algorithm has been made freely available by the authors at the following URL: http://sourceforge.net/projects/lonestar/.

**Fig. 2:**
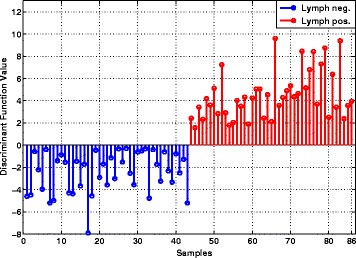
Values of the discriminant function on the training cohort of 86 tumors. Negative values of the discriminant correspond to labelling the tumor as node-negative, while positive values of the discriminant correspond to labelling the tumor as node-positive. The 43 node-negative tumors are on the *left side* of the plot, and the 43 node-positive tumors are on the *right side* of the plot. It can be seen that the discriminant values of all node-negative tumors are negative, and that the discriminant values of all node-positive tumors are positive. Thus the classifier achieves 100% accuracy on the training cohort

To detect discriminatory features that may predict metastatic disease, 213 miRNA expression features measured in 86 samples (43 lymph node-positive and 43 lymph node-negative) were used as the training data after normalization (Additional file 1: Table S3). The application of the lone star algorithm in the training data with 80 random cross validations at each iteration resulted in a set of 18 features. Afterwards, to compute a unique classifier, a single iteration of lone star is run with these 18 features and the 20 best-performing classifiers giving the best cross-validation error were computed (Additional file 1: Table S4). To have a more robust classifier the weight vectors and thresholds of these 20 classifiers were averaged to arrive at the weight vector and threshold of the final classifier. Table 2 gives the details of the classifier, including the 18 miRNAs, the weights assigned to their expression levels, and the threshold. This classifier was applied to the 86 tumor training cohort, and it classified all 86 tumors correctly. Figure 2 shows the values of the discriminant function on the expression levels of all 86 tumors.

**Table 2:** Micro-RNA signature

Micro-RNA	Weight
hsa-miR-3607-3p	–2.43
hsa-miR-299-5p	2.01
hsa-miR-365	1.747
hsa-miR-513a-5p	–2.4368
hsa-miR-29b-1*	2.2202
hsa-miR-340	–1.4319
hsa-miR-1284	1.8007
hsa_SNORD6	1.7312
hsa-miR-934	–2.223
hsa-miR-3182	1.8238
hsa-miR-1908	–1.1631
hsa-miR-155	–1.5283
hsa-miR-23c	1.3968
hsa-miR-451	–1.2663
hsa-miR-300	–1.4832
hsa-miR-223	1.0996
hsa-miR-150	–0.7774
hsa-miR-3613-3p	1.3349
Threshold	–1.0025

### Biological significance of selected biomarkers

**Fig. 3:**
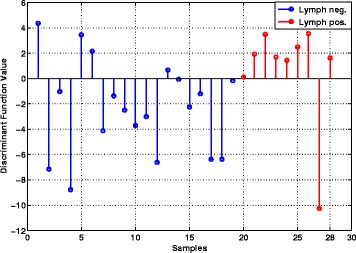
The network of 740 genes regulated by the 18 micro-RNA features. The micro-RNA with the vast majority of interactions, which are all confirmed, is hsa-mir-155. Out of the 18 micro-RNAs, three are differentially expressed across the two classes (lymph-positive and lymph-negative) in the training cohort of 86 tumors. The genes regulated by these three micro-RNAs are also shown in the figure

**Fig. 4:**
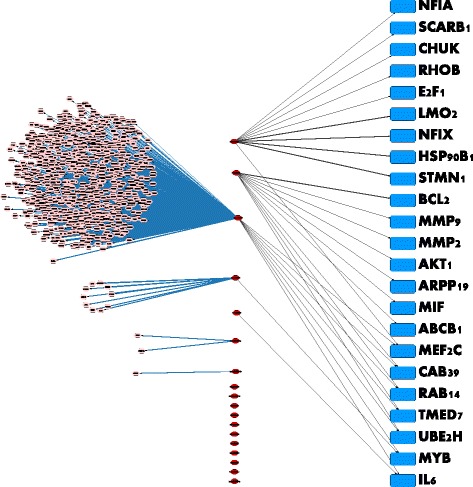
The Set of 23 Key Genes and Their Controlling micro-RNAs. Genes in this figure satisfy one of two criteria: (i) The gene is targeted by more than one micro-RNA in the set of 18 features, or (ii) the gene is targeted by one of the three differentially expressed micro-RNAs, which are the first three, namely hsa-mir-223, hsa-mir-451, and hsa-mir-155

We next carried out an analysis of the various genes that are regulated by the 18 miRNAs in the final feature set. The results are shown in Table 3. We retrieved data from the miRTarbase database, which comprises experimentally validated micro-RNA to target gene interactions in humans. A total of 740 genes were recovered, the vast majority of which are associated with the micro-RNA hsa-mir-155. A recent study suggests that hsa-mir-155 is over-expressed in endometrial cancer patients vis-a-vis normal patients [25]. We next computed the average expression value of each of the 18 miRNAS within the 43 node-positive samples as well as the 43 node-negative samples to identify those with a statistically significant differential representation between node positive versus node negative tumors. This returned hsa-miR-340, hsa-miR-451, hsa-miR-1284, has-miR-1908 and hsa-miR-223 (*P*<0.05, student *t*-test). To prune the list of 740 miRNA targets, we used two criteria: (i) A gene is targeted by more than one microRNA in the set of 18 features, or (ii) A gene is targeted by one of the five differentially expressed microRNAs. This reduced the number of genes to 23. Note that out of the five differentially expressed miRNAs only hsa-miR-223 and hsa-miR-451 have known experimentally validated targets. The resulting networks are shown in Figs. 3 and 4.

**Table 3:** The list of 23 genes and associated cancer sites

Gene	Associated cancer sites
BCL2	Colorectal cancer, Small cell lung cancer, Prostate cancer
MMP2	Bladder cancer
E2F1	Non-small cell lung cancer, Pancreatic cancer, Small cell lung cancer, Prostate cancer, Bladder cancer
MMP9	Bladder cancer
AKT1	Endometrial cancer, Colorectal cancer, Acute myeloid leukemia, Non-small cell lung cancer, Pancreatic cancer, Small cell lung cancer, Prostate cancer
HSP90B1	Prostate cancer
CHUK	Acute myeloid leukemia, Pancreatic cancer, Small cell lung cancer, Prostate cancer
IL6	Prostate cancer
NFIA	
SCARB1	
RHOB	
LMO2	
NFIX	
STMN1	
ARPP19	
MIF	
ABCB1	
MEF2C	
CAB39	
RAB14	
TMED7	
UBE2H	
MYB	

Next, we compared the list of 23 genes to the pathways in the KEGG database. Several cancer pathways were examined, and for each pathway, the *q*-value of the gene set was computed. The *q*-value is obtained from the Fisher exact test after the Benjamini-Hochberg multiple testing correction and quantifies the statistical significance of the overlap between the gene list and a set of genes in a particular pathway. The complete list of pathways examined and the associated *q*-values were computed (Additional file 1: Table S10). The most enriched pathways are Prostate Cancer, Small Cell Lung Cancer and Bladder Cancer with *q*-values of 0.00074, 0.01048 and 0.01418 respectively.

### Classifier validation with an independent cohort

To rigorously test the classifier developed using the lone star algorithm, an independent cohort of primary tumors with known metastatic state was collected. This comprised 28 endometrial cancer samples obtained between 2010 and 2012 under an IRB approved Comprehensive Gynecologic Oncology Tumor Repository protocol. Patients were consented according to protocol and fresh tumor was obtained in the operating room after the uterus was excised from the patients and bivalved with a scalpel. Tissue was flash frozen in liquid nitrogen and stored at −80°. The cohort included 19 corpus confined endometrial cancers and 9 metastatic endometrial cancers (Additional file 1: Table S5). Eight cases in the latter group demonstrated nodal metastasis while one (sample 198) had metastatic disease involving the left fallopian tube. Six sampled lymph nodes from this patient were negative for metastatic disease. However, this patient developed recurrent disease involving the left lung within six months of completing adjuvant chemotherapy. Thus the surgeons believe that this patient actually presented with metastatic disease that was not detected.

**Fig. 5:**
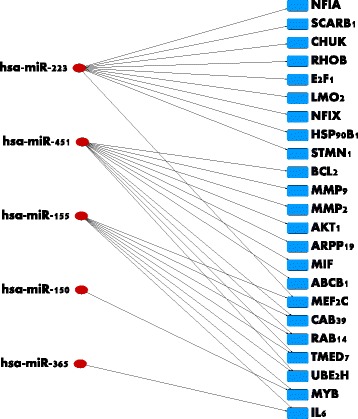
Values of the discriminant function on the independent cohort of 28 tumors. Negative values of the discriminant correspond to labelling the tumor as node-negative, while positive values of the discriminant correspond to labelling the tumor as node-positive. The 19 node-negative tumors are on the *left side* of the plot, and it can be seen that 15 out of 19 tumors have negative discriminant values and are thus classified correctly. The 9 node-positive tumors are on the *right side* of the plot, and it can be seen that 8 out of 9 tumors have positive discriminant values and are thus classified correctly

MicroRNAs were extracted and measured using the identical procedures as described for the training cohort with the exception that the Exiqon version 6 arrays were replaced by version 7 (Additional file 1: Table S9). For all of the 28 samples the discriminant value is calculated using the classifier obtained from the training data. The discriminant values for each of the 28 samples are given in the Additional file 1: Table S11 and plotted in Fig. 5. The left-most nineteen samples correspond to lymph node-negative patients while the right-most nine samples are lymph node-positive patients. A patient with a positive discriminant value is predicted to be lymph positive, while a patient with a negative discriminant value is predicted to be lymph negative. Sample 198 is the right-most point in each plot. It can be seen that the value of the discriminant function is very large for this sample. This reinforced our suspicion that the clinical annotation of this sample as lymph node-negative is erroneous and that in fact this patient had metastatic disease.

The quality of the classification results were determined with a 2×2 contingency table, and computing the likelihood of arriving at the classifications purely through chance. *P*-values were computed using the Fisher exact test [26] and the Barnard exact test [27] (Table 4). When sample 198 was treated as being lymph node-positive, as potentially justified by the fact that the patient subsequently developed recurrent disease within the left lung, the *P*-value was 0.0004 with the Barnard exact test, and 0.0012 with the less powerful Fisher exact test. Even when sample 198 was treated as lymph node-negative, and thus as having been missclassified by the classifier, the classification had a *P*-value of 0.0037 with the Barnard exact test and 0.0107 with the Fisher exact test. In the other direction, the false negative likelihood of this classifier was 1/16=0.0625 when sample 198 is treated as node-positive. In other words, among the 16 patients classified as being not at risk for lymph node metastasis, only one patient was actually at risk.

**Table 4:** Contingency table of classifier performance on test cohort

Actual/Classification	Positive	Negative	Total	Positive	Negative	Total
Node-Positive	8	1	9	7	2	9
Node-Negative	4	15	19	4	15	19
Total	12	16	28	11	17	28
Accuracy	0.8214			0.7857		
Sensitivity	0.8889			0.7778		
Specificity	0.7895			0.7895		
False Discovery Rate	0.0625			0.1174		
*P*-Value (Fisher)	0.0012			0.0104		
*P*-Value (Barnard)	0.0004			0.0037		

(The performance of the classifier on the 86 training cohort is not shown as it was 100%.) The left part of the table corresponds to sample #198 treated as node-positive, while the right part of the table corresponds to sample #198 treated as node-negative. When sample #198 is treate as node-positive, the classifier has accuracy of 82.14%, with 23 out of 28 tumors being correctly classified; sensitivity of 88.89% with 8 out of 9 lymph-positive tumors being correctly classified; and specificity of 78.95%, with 15 out of 19 lymph-negative tumors being correctly classified. The *P*-value of obtaining these values purely by chance was computed using the Fisher exact test at 0.0012 and as 0.0004 using the more powerful Barnnard exact test. The corresponding figures with sample #198 treated as node-negative are shown for comparison. It can be seen that even this case, all *P*-values are far lower than the widely accepted threshold of 0.05

## Discussion

### Lone star as a sparse classification algorithm

The development of the support vector machine (SVM) [10] was a major milestone in machine learning, because the algorithm is very robust numerically, and can therefore handle very large datasets. The original SVM formulation was for engineering problems, where it is relatively easy to generate a large number of samples, as a result of which the number of features is far smaller than the number of samples. However, it was recognized almost at once that the standard SVM formulation had some weaknesses when applied to biological datasets, where the situation is the reverse. Specifically, the classifier produced by the traditional SVM assigns a nonzero weight to every single feature. When the number of features is larger than the number of samples, this leads to a phenomenon known as “over-fitting," wherein a classifier does an excellent job on the training data, but has poor generalization abilities. This phenomenon is also referred to as “memorization" of the training data.

To overcome this shortcoming, several approaches have been proposed in the literature. The *ℓ*_1_-norm SVM of [11] suggests replacing the traditional *ℓ*_2_-norm proposed in [10] with the *ℓ*_1_-norm, which is the sum of the absolute values of the components of a vector. As shown in [11], the *ℓ*_1_-norm SVM is guaranteed to choose no more features than the number of samples, no matter how large the number of features happens to be. However, when the number of samples is around a hundred, as in the application studied in the present paper, even this number is too large to be useful in practice. An entirely different approach known as recursive feature elimination is proposed in [14], in which a traditional (*ℓ*_2_-norm) SVM is trained, the feature with the smallest weight (in magnitude) is dropped, and the algorithm is run anew. In [14] the approach was applied to a leukemia data set, and identified just two features as being sufficient. However, in general, the performance of the algorithm is non-monotonic, meaning that the performance on training data tends to go up and down as more and more features are dropped. For this reason, the recursive feature elimination step needs to be repeated many times from different starting points. Another improvement in machine learning is stability selection proposed in [15], that suggests running an algorithm many times with different random partitionings of the available data into training and testing datasets. Stability selection ensures that the finally selected feature set is quite robust against measurement noise. The lone star algorithm combines the above-mentioned ideas in a self-contained package. Specifically, the objective function minimized in the lone star algorithm is a convex combination of the *ℓ*_1_- and the *ℓ*_2_-norms. In this respect, the algorithm differs from both the *ℓ*_2_-norm SVM of [10] as well as the *ℓ*_1_-norm SVN of [11]; it also differs from the Elastic Net formulation of [12]. In addition, the algorithm also incorporates differential weighting for false positive and false negatives [28] and an optional *t*-test to filter the features when their initial number is very large. Our new algorithm is therefore of interest to the theoretical machine learning community.

While the above-mentioned ideas have been individually proposed in the machine learning literature, thus far they have not been effectively combined into one algorithm. The closest approach to the lone star algorithm is the so-called SVM-T-RFE algorithm introduced in [29]. In that algorithm, the authors use as their starting point the SVM-RFE approach suggested in [14], and also compute the *t*-test statistic to determine whether an individual gene does, or does not, show a significant variation between the two classes to be discriminated. Thus the SVM-T-RFE algorithm in [29] still uses the traditional SVM formulation based on Euclidean distances, which causes all genes to be assigned positive weights in general. Then a new figure of merit is computed for each gene, which is a combination of its weight from the Euclidean norm-based SVM output and the *t*-test statistic. The gene (or feature) with the smallest of merit is discarded, and the process is repeated. This is in contrast to the lone star algorithm, wherein a combination of the *ℓ*_2_- and the *ℓ*_1_-norm distance measures is used, which causes most weights to exactly equal zero. As a result, a large number of features can be eliminated at each iteration, as opposed to one feature at a time in SVM-T-RFE. Consequently the lone star algorithm converges far more quickly and is also more numerically stable, compared to SVM-T-RFE and other methods based on using Euclidean distance measures.

### Application to endometrial cancer

The problem of assessing the risk of endometrial cancer patients for lymph node metastasis has been the subject of much study over the years. So far as we are able to determine, the present study is one of only two in which predictive biomarkers were tested with an independent sample cohort, the other being [30]. Validation on an independent cohort is vital to determine whether the prediction methodology is robust against unavoidable variations in measurement platforms and experimental protocols. If a prediction methodology is cross-validated on a common cohort, all of the potential variations in data introduced by platform-and protocol-dependencies are absent. This can lead to misleadingly high performance that may or may not be repeated with a genuinely independent data set.

The ultimate objective of a molecular signature for endometrial cancer should be to identify patients who are not at risk of lymph node metastasis, in such a way that most patients who require lymphadenectomy receive it. However, in every clinical test there is an associated false negative rate and a good test should be able to make this rate acceptably small, say around 5%. In the validation analysis presented here, 8 out of the 9 surgically confirmed node positive patients were correctly identified. In addition, 15 out of the 16 patients classified as not requiring surgery were surgically confirmed node negative patients. Thus the classifier achieved both desired objectives within a significant confidence interval. Application to much larger patient cohorts is anticipated to determine if appropriate receiver operator characteristics can be achieved for clinical application as a diagnostic.

## Conclusions

In this work, we have developed a novel sparse classification algorithm and applied it to predict risk of lymph node metastasis in endometrial cancer patients. The algorithm produced a weighted classifier, using 18 micro-RNAs, and achieved 100% accuracy on the training cohort. When applied to an independent testing cohort, the classifier correctly predicted 90% of node-positive cases, and 80% of node-negative cases (FDR =6.25*%*).

The classifier developed in this study was based on molecular measurements from excised tumors. If one could predict the risk of lymph node metastasis on the basis of a biopsy, then the decision to carry out lymphadenectomy or not could be made at the time of excision of the primary tumor. Therefore a useful next step would be to repeat the present study on a cohort of biopsies. Pending the completion of such a study, it is worth noting that a prediction of the risk of metastasis is valuable even if lymphadenectomy is not performed, as it can inform choices for post-resection patient care.

## Methods

### Selection of specimens

Fifty stage I and 50 stage *IIIC* frozen endometrioid endometrial cancer samples were obtained from the Gynecologic Oncology Group (GOG) tumor bank. The samples were collected from patients enrolled in GOG tissue acquisition protocol 210 which established a repository of clinical specimens with detailed clinical and epidemiologic data from patients with surgically staged endometrial carcinoma. All patients enrolled in GOG 210 have undergone comprehensive surgical staging consisting of total abdominal hysterectomy, bilateral salpingo-oophorectomy, pelvic and para-aortic lymphadenectomy. While there was no mandated minimum lymph node count for inclusion on GOG protocol 210, specific procedural requirements for pelvic and para-aortic lymphadenectomy were stipulated which necessitated removal of all lymphatic tissue from the relevant lymphatic beds. Patients included in our study had no gross or pathologic evidence of extra-uterine disease and could be considered clinical stage I tumors. All tumors have undergone central pathologic review by the GOG and contain ≥75*%* tumor.

### MicroRNA isolation and array analysis:

Once the tumor samples were collected, frozen tissue was added to a chilled BioPulverizer H tube (Bio101, Irvine, CA). Lysis buffer from the Ambion mirVana microRNA isolation kit (Ambion, Austin TX) was added and the tissue homogenized for two minutes in a Mini-Beadbeater (Biospec Products, Bartlesville, OK). Tubes were spun briey to pellet the garnet mixture and reduce foam. The lysates were then transferred to a new 1.5 ml tube using a syringe. MicroRNA was then extracted using the Ambion mirVana microRNA isolation kit (Ambion, Austin TX).

### Array methods:

Total RNA samples were labeled with Hy3 using the Hi-Power labeling kit (Exiqon) per the manufacturers protocol. miRCURY LNA microRNA Array Spike-in kit v2 (Exiqon) was used as a control for the labeling reaction and to calibrate scanner settings. Briefly, 1.5 *μ*g total RNA in 3 *μ*L,1 *μ*L spike-in miRNA kit v2, 0.5 *μ*L CIP buffer and 0.5 *μ*L CIP enzyme were mixed on ice and incubated at 37° for 30 minutes. The RNA was then denatured at 95° and then immediately placed on ice for at least 2 minutes. This reaction product was then mixed with 3 *μ*L Hi-Power labeling buffer, 1.5 *μ*L Hy3 uorescent label, 2 *μ*L DMSO and 1 *μ*L Hi-Power labeling enzyme for a total of 12.5 *μ*L, and then incubated for 2 hours at 16°. Samples were subsequently hybridized to microarray slides (Exiqon miRCURY LNA microRNA 6th generation array) using a NimbleGen/MAUI 4-Bay hybridization station per the manufacturers protocol. Briefly, the labeled RNA was brought up to 25 *μ*L volume and 25 *μ*L of hybridization buffer (Exiqon) was added. This solution was then denatured at 95° and put on ice for at least 2 minutes. Microarray slides were placed in hybridization chambers and pre-warmed to 56° for at least 5 minutes. A total of 45 *μ*L of sample was added to the microarray slide and hybridized for 16 hours at 56° in the hybridization chamber. Slides were then washed once for 2 minute at 56° in wash buffer A (Exiqon) and once for 2 min at 23° in wash buffer B (Exiqon). Slides were then washed for 2 minutes at 23° in wash buffer C (Exiqon), washed briey in 99% ethanol, and then spun in a centrifuge (1000 rpm) for 5 minutes to dry. Microarray slides were scanned using the Tecan PowerScanner scanning system. Spot quantification and statistical analysis were performed using ImaGene 9 and Nexus Expression 2 software (BioDiscovery Inc.) using the Exiqon default settings. Briefly, for quality control, correlation coefficients of spike-in controls across arrays were calculated, and arrays with correlation coefficients less than 0.8 were removed from the dataset; spot background subtraction was done by subtracting the median local background from the mean intensity of the spot, replicated probes on each array were combined into one output value using the median value, and normalized across all arrays using quantile normalization. The data output was log 2 transformed.

### Lone star algorithm:

Suppose we are given a set of labeled data here $x^{i} \in \mathbb {R}^{n}$ and *y*_*i*_∈{−1,1} for *i*=1,2,⋯,*m*. Therefore, *n* denotes the number of features and *m* denotes the number of samples. The feature vector *x* ^*i*^ is viewed as a row vector. The objective is to choose a subset of features *F*⊆{1,⋯,*n*}, a weight vector $w \in \mathbb {R}^{n}$ and a threshold $\theta \in \mathbb {R}$ such that

(a) the discriminant function *f*(*x*_*i*_)=*x*_*i*_ *w*−*θ* has the same sign as *y*_*i*_ for most indices *i*,

(b) *w*_*j*_=0 for all *j*∉*F*, and (c) |*F*|≪*m*.

In words, the discriminant function *f* is linear, and the set of features used by the discriminant has smaller cardinality than the number of samples. Define

$$P=\{i:y_{i}=1\},N=\{i \in y_{i} = -1\}. $$

and let *m*_1_=|*P*|, *m*_2_=|*N*|. The algorithm consists of three parts, namely: an optional preprocessing step, an iterative loop and a final classifier generation step. The first step is the preprocessing, steps 2 through 4 are the iterative loop, and step 5 is the final classifier generation.

1. **Normalization of Feature Vectors:** Normalize each of the remaining feature vectors by subtracting the mean over all *m* samples and then scaling so that the resulting vector has Euclidean norm of one. The resulting vector is just the set of Z-scores divided by $\sqrt {m}$. Set the iteration counter to 1, the feature set *F* to the set of significant features, the feature count *s*_1_ to |*F*|, the iteration count *i* to one, and proceed to the iterative loop.
2. **Stability Selection:** Fix an integer *l*. Choose at random *k*_1_ out of the *m*_1_ positive samples and *k*_2_ out of the *m*_2_ negative samples as the “training" set of samples. Repeat this random choice *l* times, so that there are *l* different pairs of training samples: *k*_1_ from the class *P* and *k*_2_ from the class *N*. Ensure that *k*_1_ and *k*_2_ are roughly equal and roughly equal to the smaller of *m*_1_/2,*m*_2_/2.
3. **Combined** ***ℓ***_**1**_ **- and** ***ℓ***_**2**_ **-Norm SVM:** For each pair of *k*_1_,*k*_2_ training samples, solve the following *ℓ*_1_-norm support vector machine formulated in [11]:
  $$\begin{aligned} &{\min_{\mathbf{w}, \theta, \mathbf{y}, \mathbf{z}}} (1 - \lambda) \left[ \alpha \sum_{j=1}^{k_1} y_{j} + (1 - \alpha) \sum_{j=1}^{k_2} z_{j} \right] \\ &+ \lambda \left[ \gamma \sum_{i=1}^{s} | w_{i} | + (1 -\gamma) \left(\sum_{i=1}^{n} w_{i}^{2} \right)^{1/2} \right], \end{aligned} $$
  subject to the constraints
  $$\mathbf{w}^{t} \mathbf{x}_{j} - \theta + y_{j} \geq 1, j \in P, \mathbf{w}^{t} \mathbf{x}_{j} - \theta - z_{j} \leq -1, j \in N, $$
  $$\mathbf{y} \geq \mathbf{0}_{k_1}, \mathbf{z} \geq \mathbf{0}_{k_2}. $$
  The parameter *λ* should be chosen “close to" zero but not exactly zero. The parameter *α* should be chosen as 0.5 if sensitivity and specificity are equally important. To place more emphasis on sensitivity, *α* should be chosen less than 0.5, while *α* should be chosen to be greater than 0.5 to place more emphasis on specificity. Finally the parameter *γ* adjusts the relative weights given to the *ℓ*_1_- and *ℓ*_2_-norms. In this study, we choose *α*=0.5 and we used 2-fold cross-validation for tuning *γ*=0.5.
4. **Recursive Feature Elimination (RFE):** The previous step results in *l* different optimal weight vectors $w_{1}^{i},\cdots,w_{l}^{i}$, where *i* is the iteration count. Each weight vector will have a different number of nonzero components. Compute the average number of nonzero components, and round upwards to the next integer. Denote this integer as *r* ^*i*^. Compute the average of all *l* weight vectors. Retain the *r* ^*i*^ components with the largest magnitude and discard the rest. Increment the iteration counter *i*, set *s* ^*i*+1^=*r* ^*i*^, and proceed to Step 3. If *R* ^*i*^=*s* ^*i*^, meaning that no features can be discarded, the iterative step is complete. Proceed to the next step.
5. **Final Classifier Generation:** When this step is reached, the set of features is finalized. Run the *ℓ*_1_-norm SVM on *l* different randomly chosen pairs of (*k*_1_,*k*_2_) training samples to generate *l* different classifiers and evaluate the performance of each of the *l* classifiers on the remaining (*m*_1_−*k*_1_,*m*_2_−*k*_2_) samples. Determine the accuracy, sensitivity, and specificity of each of the *l* classifiers. Average the weights and thresholds of the best-performing classifiers to generate an overall classifier.

## Additional file

Additional file 1 List and description of supplemental tables. **Table S1.** This table contains the measurements of 1428 micro-RNAs for 94 Samples. The rows correspond to the features (miRNA) and the columns correspond to the samples. The samples consist of 47 lymph node-positive and 47 lymph node-negative samples. 43.75% of the entries in this sheet are NaN. It contains measurements for 213 miRNAs of 86 samples. Out of those 86 samples, 43 are lymph node-positive, and the remaining 43 are lymph node-negative. A sample whose label has the term IB or IC belongs to a lymph node-negative patient, whereas a sample with a label containing IIIC belong to a lymph node-positive patient. A lymph node-positive or neagtive status was defined empiracally during pimary staging. **Table S2.** This table contains a subset of the raw data, used for training the classifier. This data was obtained by removing four patients from each class, and 1,215 features. It contains measurements for 213 miRNAs of 86 samples. Out of those 86 samples, 43 are lymph node-positive, and the remaining 43 are lymph node-negative. **Table S3.** This table contains the normalized version of the training data. The following procedure is used for normalization: 1) From each entry of the *i*-th row vector (*i*-th feature vector), we subtract the mean value *m*_*i*_ of the *i*-th row vector computed over all the 86 samples. 2) Multiply each entry of the *i*-th row vector by a scale factor *s*_*i*_ so that the resulting vector has euclidean norm equal to the square root of 86. **Table S4.** The lone star algorithm selected 18 final features. This sheet contains the 20 best classifiers based on these eightteen features, sorted with respect to accuracy. The sensitivity, specificity and accuracy figures (columns T, U and V) are based on the classification of the 86 samples in the training data by the corresponding classifier.**Table S5.** This table shows the classifier obtained by taking the average of the classifiers in Sheet 4. In particular, we average the numbers in each column of the 20 classifiers given in Sheet 4 (20 best classifiers) (Columns A-S). **Table S6.** This sheet contains clinical information about the independent cohort of 28 patients who were used to validate the classifier. Out of these, 9 are lymph-node positive and 19 are lymph node-negative. **Table S7.** This sheet contains the raw microRNA measurements on the 28 test data samples. **Table S8.** This is the transformed version of the test data. We apply the same transformation as w did for the training data, as described on Sheet 3. For each of the 18 features (miRNAs), we subtract the original mean value *m*_*i*_ from each entry and multiply each entry by the constant *s*_*i*_. The calculation of *m*_*i*_ and *s*_*i*_ is as in Additional file 1, Table S3. **Table S9.** This sheet contains the discriminant values of the classifier on the Test Data. In column D an entry of 1 means that the sample is correctly classified. **Table 10.** This sheet contains the number of overlaps between our 23 gene signature with the pathways in the KEGG database. The *q*-value is obtained from the Fisher exact test after the Benjamini-Hochberg multiple testing correction and quantifies the statistical significance of the overlap between the gene list and a set of genes in a particular pathway. (1170 KB XLSX)
